# Enhancing Genome-Wide Copy Number Variation Identification by High Density Array CGH Using Diverse Resources of Pig Breeds

**DOI:** 10.1371/journal.pone.0087571

**Published:** 2014-01-27

**Authors:** Jiying Wang, Jicai Jiang, Haifei Wang, Huimin Kang, Qin Zhang, Jian-Feng Liu

**Affiliations:** 1 Key Laboratory of Animal Genetics, Breeding and Reproduction, Ministry of Agriculture, National Engineering Laboratory for Animal Breeding, College of Animal Science and Technology, China Agricultural University, Beijing, China; 2 Shandong Provincial Key Laboratory of Animal Disease Control and Breeding, Institute of Animal Science and Veterinary Medicine, Shandong Academy of Agricultural Sciences, Jinan, China; Wageningen UR Livestock Research, Netherlands

## Abstract

Copy number variations (CNVs) are important forms of genomic variation, and have attracted extensive attentions in humans as well as domestic animals. In the study, using a custom-designed 2.1 M array comparative genomic hybridization (aCGH), genome-wide CNVs were identified among 12 individuals from diverse pig breeds, including one Asian wild population, six Chinese indigenous breeds and two modern commercial breeds (Yorkshire and Landrace), with one individual of the other modern commercial breed, Duroc, as the reference. A total of 1,344 CNV regions (CNVRs) were identified, covering 47.79 Mb (∼1.70%) of the pig genome. The length of these CNVRs ranged from 3.37 Kb to 1,319.0 Kb with a mean of 35.56 Kb and a median of 11.11 Kb. Compared with similar studies reported, most of the CNVRs (74.18%) were firstly identified in present study. In order to confirm these CNVRs, 21 CNVRs were randomly chosen to be validated by quantitative real time PCR (qPCR) and a high rate (85.71%) of confirmation was obtained. Functional annotation of CNVRs suggested that the identified CNVRs have important function, and may play an important role in phenotypic and production traits difference among various breeds. Our results are essential complementary to the CNV map in the pig genome, which will provide abundant genetic markers to investigate association studies between various phenotypes and CNVs in pigs.

## Introduction

Pigs have been one of the most economically important livestock worldwide for over 10,000 years as an important animal supplying meat [1]. Pigs are also closely related to humans in terms of anatomy, genetics and physiology, and represent an excellent animal model to study various human diseases [2], [3]. Because of the economic and medical importance of pigs, the Swine Genome Sequencing Consortium (SGSC) has decoded swine whole-genomic information. The porcine genome consists of 18 autosomes and sex chromosomes with a genome size estimated to be around 2.8 Gbp and a minimum of 21,640 protein-coding genes involved [4]. The completion of porcine genome and its annotation, together with chips and high throughput sequencing technologies, make it possible to study the genomic variations in-depth.

So far a wide range of genomic variations have been found existing across genome, from single nucleotide polymorphisms (SNPs) to structure variations with sizes ranging from kilobases (Kb) to megabases (Mb). As a major form of genomic variations, copy number variations (CNVs) are defined as gains and losses of genomic sequence greater than 50 bp between two or more individuals of a species [5], [6]. Compared with the most frequent polymorphisms of SNPs, CNVs cover wider genomic regions in terms of total bases involved and have potentially larger effects by changing gene structure and dosage, alternating gene regulation, exposing recessive alleles and other mechanisms [7], [8]. In humans, since the milestone works by Iafrate et al. and Sebat et al. 2004 [9], [10], CNVs have attracted extensive attentions and 109,863 CNVs have been identified (http://dgv.tcag.ca/dgv/app/, July 2013). Studies in domestic animals have shown that a suite of genes with copy number alteration were found contributing to variation of either phenotypic variability or disease susceptibility, such as the *KIT* gene for white coat phenotype in pigs [11], *SOX5* gene for the pea-comb phenotype in chickens [12], *STX17* gene for hair greying and susceptibility to melanoma in horses [13]. Additionally, the study by Seroussi et al. [14] indicated there were close associations between CNVR, located on BTA18, and index of total merit and genetic evaluations for protein production, fat production and herd life in Holstein cattle.

Using different technological platforms, substantial progress has been made in identifying CNVs in pigs. For example, based on Porcine SNP60 BeadChip and aCGH, Ramayo-Caldas et al. [15], Wang et al. [16], [17], Chen et al.[18], Fadista et al. [19] and Li et al.[20] identified hundreds of CNVRs. Recently, based on genome re-sequencing, Rubin et al. [21] and Paudel et al. [22] also detected a large amount of CNVs. However, compared to humans and other model organisms, relatively few studies have investigated CNVs in pigs and little is known about how CNVs contribute to normal phenotypic variation and to disease susceptibility in this species. Findings from previous studies also indicate that besides the platforms employed in CNV detection, a considerable proportion of CNVs segregate among distinct breeds or populations [6], [18], [23]. Hence, a sufficient high-resolution CNV map requires the survey of multiple breeds/populations. Chinese indigenous breeds have larger genetic diversity and higher average heterozygosity than European breeds [24], which can help to detect fruitful breed-specific CNVs which have segregated among different populations in the course of evolution and selection.

To comprehensively identify genome-wide CNVs across diverse pig breeds, in the present study, one custom-designed high-density genome-wide tiling aCGH (2.1 M) based on the newest build of porcine genome *Sscrofa* 10.2 [4] was used to detected CNVs among 12 test samples, which were selected from diverse populations, including six types of Chinese indigenous breeds, one Asian wild boar population, as well as two modern commercial breeds. Consequentially, we identified a large amount candidate CNVRs, which are essential complementary to the CNV map in the pig genome.

## Materials and Methods

### Ethics statements

The whole procedure for collection of the ear tissue samples of all animals was carried out in strict accordance with the protocol approved by the Institutional Animal Care and Use Committee (IACUC) of China Agricultural University.

### Selection of pig breeds and animals

In the present study, 12 individuals, selected from diverse populations, were used as test samples, while one Duroc was used as the reference sample. These 12 animals include one wild pig, two pigs each from Yorkshire and Landrace as the representatives of modern commercial breeds and nine unrelated individuals selected from six Chinese indigenous breeds (2- Tibetan pig, 2- Diannan small-ear pig, 2-Meishan pig, 1-Min pig, 1-Daweizi pig, and 1-Rongchang pig). According to the geographic distribution and phenotypic features, the existing Chinese indigenous pig breeds have been divided into six distinct population types [25]. The six local breeds investigated each being selected from a specific population type are considered as the representatives of Chinese local population, which are Tibetan pig (Plateau Type), Diannan small-ear pig (South Chine Type), Meishan pig (Lower Changjiang River Basin Type), Min pig (North China Type), Daweizi pig (Central China Type), and Rongchang pig (Southwest Type) respectively.

Genomic DNA for each of 13 individuals was extracted from the ear tissue using Qiagen DNeasy Tissue kit (Qiagen, Germany). The concentration and its quality of each total genomic DNA were determined with NanoDrop (NanoDrop Technologies, Wilmington, DE, USA) and 1% agarose gel electrophoresis.

### Array CGH design, hybridization and CNV calling

One genome-wide 2.1 M aCGH was designed and produced by NimbleGen (Roche NimbleGen, Inc., Madison, WI, USA) based on the newest build of porcine genome (*Sscrofa* 10.2) (http://www.animalgenome.org/repository/pig/). This array covered 18 autosomes and two sex chromosomes, and contained 2,167,769 oligonucleotide probes (50–75 mers), with a median and average intervals of 900 bp and 889 bp, respectively. The sample of Duroc was used as the reference, while the other 12 individuals as the test samples in the experiment. Genomic DNA labeling, hybridization and array scanning were performed according to the manufacturer's instructions.

Spatial correction and data normalization, segmentation were performed using DEVA 1.2 software (Roche-NimbleGen). Specifically, locally weighted polynomial regression (LOESS) was used to adjust signal intensities based on X, Y feature position. Normalization was then performed using the q-spline method followed by segmentation using the CNV calling algorithm segMNT included in DEVA 1.2. To call high-confidence CNVs, two criteria, *i.e.*, mean log_2_ ratio ≥|0.5| and at least 5 consecutive probes, were used to filter CNVs. Since the CNV calling pipeline requires at least 5 consecutive probes, our theoretical resolution for CNV detection is 3,875 bp (median spacing ×4 +median oligo length ×5). Finally, CNVRs were determined by aggregating overlapping CNVs identified across all samples according to the criteria previously described [26]. The raw data of our custom-designed aCGH experiments and the sequence information of our probes have been deposited into the GenBank GEO database (GSE46847) (http://www.ncbi.nlm.nih.gov/geo/query/acc.cgi?acc=GSE46847).

### False positive rate

In the study, two of the 12 test samples, DN1 and R2, were male, while the reference, Duroc, was female. Based on the two sex-mismatched arrays, we assessed the false positive rate produced under the criteria using the similar method as reported previously [27]. Specifically, the false positive rate was calculated by the length of chrX having a log_2_-ratio with a different signal than it should (given the sex-mismatched hybridization) divided by the length of chrX ((3,149,134+1,496,534)/(2*144,288,218)  = 1.61%).

### Quantitative real time PCR (qPCR) confirmation

qPCR was used to validate 16 CNVRs identified by aCGH array. The glucagon gene (GCG) is highly conserved between species and has been approved to have a single copy in animals [28], and one segment of it was chosen as the control region. Primers were designed for the target and control regions with the Primer3 web tool (http://frodo.wi.mit.edu/primer3/). Moreover, the UCSC In-Silico PCR tool (http://genome.ucsc.edu/cgi-bin/hgPcr?command=start) was used for in silico specificity analysis. Prior to performing the copy number assay, we generated standard curves for the primers of target and control regions to determine their PCR efficiencies, which were required to be 1.95–2.10 to ensure the same amplification efficiencies between target and control primers. All qPCR were carried out using LightCycler® 480 SYBR Green I Master on Roche LightCycler® 480 instrument following the manufacturer's guidelines and cycling conditions. Each sample was analyzed in duplicates. The copy number for each test region was calculated using the 2^−ΔΔCt^ method [29], which compares the ΔCt (Ct of the target region minus Ct of the control region) value of the test samples with CNV to the ΔCt of the reference sample.

### Gene content and functional analyses

Pig CNVRs were annotated using NCBI gene information (ftp://ftp.ncbi.nlm.nih.gov/gene/DATA/GENE_INFO/Mammalia/Sus_scrofa.gene_info.gz). Gene Ontology (GO) terms and Kyoto Encyclopedia of Genes and Genomes (KEGG) pathway analyses were performed with DAVID bioinformatics resources 6.7 (http://david.abcc.ncifcrf.gov/). Since only a limited number of genes in the pig genome have been annotated, we firstly converted the pig Ensembl IDs to orthologous human Ensembl IDs by BioMart (http://www.biomart.org/) before GO and pathway analyses. Statistical significance was assessed by using *P* value of a modified Fisher's exact test and Benjamini correction for multiple testing. Additionally, the dN/dS ratio compared with those human species was computed for each gene, and Wilcoxon rank-sum test was used to test the difference of dN/dS ratios between copy numbers varied genes and monomorphic ones.

We also performed the overlap analyses between CNVRs identified in the study with the reported QTL regions collected in the pig QTL database (Apr 20, 2013, (http://www.animalgenome.org/cgi-bin/QTLdb/SS/index) and human disease gene orthologs in Online Mendelian Inheritance in Man annotations (OMIM, http://omim.org/, 2013-6-19).

## Results and Discussion

### Genome-wide CNVs identified among diverse pig breeds

Using the custom-designed 2.1 M aCGH (Roche NimbleGen,), we performed CNV discovery using 12 pig samples involving one wild boar population, six Chinese indigenous breeds and 2 modern commercial breeds, Yorkshire and Landrace, with one individual of the other modern commercial breed, Duroc, as the reference. Totally, we identified 2,239 CNVs in the 12 test samples with 186.6 CNVs per individual. After merging the overlapping CNVs among different samples, a total of 1,344 CNVRs (Table S1 in File S1) were detected, covering 47.79 Mb of the pig genome and corresponding to 1.70% of the genome sequence. The length of these CNVRs ranged from 3.37 Kb to 1,319.0 Kb with a mean of 35.56 Kb and a median of 11.11 Kb.

As we did not perform self-to-self experiments, more stringent criteria with the mean log_2_ ratio ≥|0.5| and five consecutive probes were used to call high-confidence CNVs according to the previous studies [20], [23] to ensure the high rate of true positive findings. In the study, two of the 12 test samples, DN1 and R2, were male, while the reference, Duroc, was female. As the females have two chromosomes X and males only have one, male-female aCGH resulted in an excess of female signals for X-linked sequences that can be used to calibrate the threshold values and detection methods. Based on the two sex-mismatched arrays, we assessed the false positive rate produced under the criteria using the similar method as reported previously [27]. According to the theoretic inference, all segments on chromosomes X should be loss in the DN1 and R2. Our results showed that there were 24 segments (4.65 Mb) with the log-intensity ratio >0, and the false positive rate on chromosomes X was 1.61%, indicating few false positive CNVs were caused under the current criteria. It is notable that the false positive rate (1.61%) is conservatively overestimated due to the assumption that there are no CNVs in chromosome X of sex-mismatched arrays.

Compared among the 12 individuals, large difference of CNVR numbers were clearly observed among them. The number of CNVs per individual ranged from 66 (Landrace) to 499 (wild boar). In general, more CNVs per individual were identified in the individuals from Chinese indigenous breeds and wild population (202.7) than in those from modern commercial breeds (109.5). The possible reason is that the relationships between modern pigs and the reference pig (Duroc) are closer than those between Chinese pigs and the reference. It highlights the importance of using samples covering a broad representation of pig diversity.

### Pattern and distribution of CNVRs

**Figure 1:**
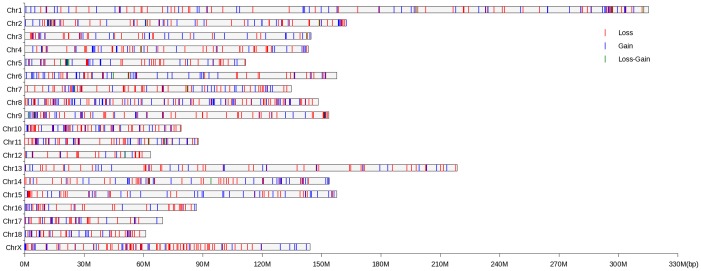
Genomic distribution of CNVRs in 18 autosomes and chromosome X of pigs. The chromosomal locations of 1,344-axis values are chromosome names, and X-axis values are chromosome position in Mb, which are proportional to real size of swine genome sequence assembly 10.2 (http://www.ensembl.org/Sus_scrofa/Info/Index).

Figure 1 illustrates the location and characteristics of all CNVRs identified across pig genome. The proportion of CNVRs on each chromosome varied from 0.75 (Chr13) to 3.33% (Chr10), with the average of 1.96%. In particular, we detected 101 CNVRs in chromosomes X with the proportion (2.43%) similar to those in the autosomes.

Concerning copy number status, the numbers of gain, loss and both events (loss and gain within the same region) were 557 (41.44%), 760 (56.55%) and 27 (2.01%), respectively. Loss events were more common than gain events in CNVRs, but had slightly smaller sizes than gains on average (29.07 Kb vs. 32.00 Kb). However, previous studies in human have suggested that losses were more deleterious than gains, and losses tended to be under stronger purifying selection than gains [30], [31]. The observation of more loss events than gain events herein is at least partially related to the technical bias. As also noted by others [26], [27], due to the CNV detection pipeline used, aCGH approach has more power to detect a loss (log_2_(1/2) = −1) than a gain (log_2_(3/2) = 0.58).

**Figure 2:**
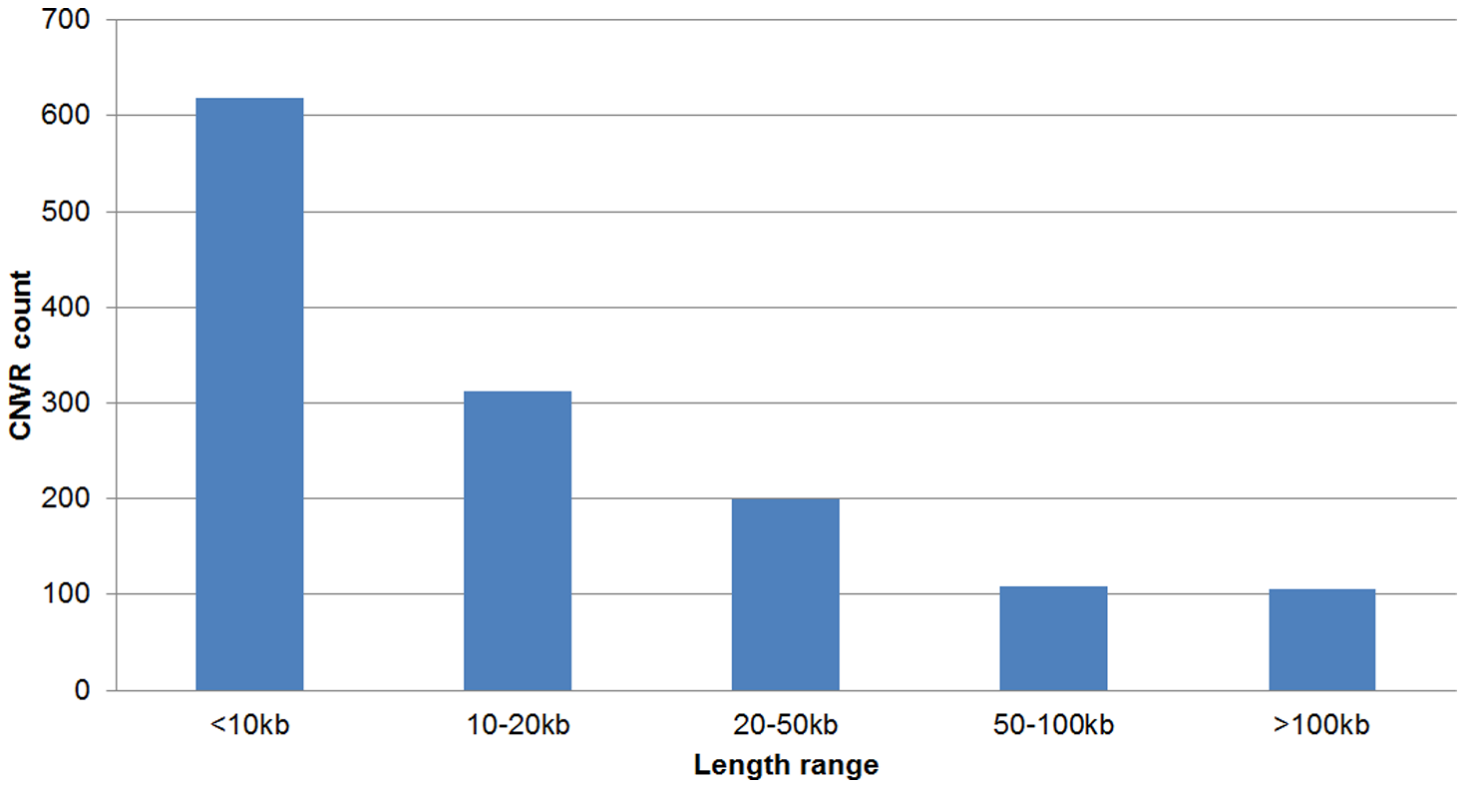
Size range distribution of CNVRs identified by aCGH.

The spectrum of the sizes of all detected CNVRs was demonstrated in Figure 2. It can be seen that almost half of CNVRs (45.98%) fell into the length interval ≤10 kb, and the number of CNVRs decreased with the increasing of the sizes. This trend was in accordance with that in humans (http://dgvbeta.tcag.ca/dgv/app/home?ref=NCBI36/hg18). In our CNV calling pipeline, CNVs were called requiring at least 5 consecutive probes with theoretical resolution of 3,875 bp. However, the resolutions of the previous swine CNV studies using aCGH, as well as porcine SNP60 BeadChip, were all larger than 10 kb. As a consequence, the current study has a better power to detect CNVs with small length.

### Comparison with previous studies

Compared with previous studies [15], [16], [18]–[22], 347 (25.82%) out of 1,344 CNVRs identified found in our study overlapped with those reported. This indicates that about one fourth of CNVRs identified in the study can be validated by previous studies, and most of our findings are first detected herein (see Table 1 and Table S2 in File S1). The most overlapped CNVR count (18.67%) and length (31.74%) were with studies of Paudel et al. [22], which detected 3,118 copy number gain events based on the genome re-sequencing data of 16 pigs. Besides it, the second overlapped CNVR length (21.69%) was with studies of Chen et al. [18], which identified 565 CNVRs in a large sample size (1,693) pigs from 18 diverse populations. The issue of low overlapping rates between different reports was also encountered in other CNV studies [20], [32], [33]. The potential reasons for the inconsistence among results of different studies lie in many aspects, such as the difference of samples in size and genetic background, different detection platforms and algorithms for CNV calling, CNV (CNVR) definition as well as potential technical and random errors.

**Table 1:** Comparison between CNVRs detected in the study with those in the previous reports.

Study	CNVR detected in the previous studies							Overlaps with this study			
	Methods	Sample	CNVR	Range (Kb)	Median (Kb)	Mean (Kb)	Total (Mb)	Count	Count Percentage	Total length (Kb)	Length Percentage
**Fadista et al., 2008 (19)**	aCGH (385 k)	12	37	1.74–61.92	6.89	9.32	0.43	1	0.07	7.37	0.02
**Ramayo-Caldas et al., 2010 (15)**	SNP chip (60 k)	55	49	44.65–10715.82	170.96	754.59	36.97	4	0.30	174.74	0.37
**Wang et al., 2012 (16)**	SNP chip (60 k)	474	382	5.03–2702.75	142.90	250.69	95.76	13	0.97	442.31	0.93
**Li et al., 2012 (20)**	aCGH (720 k)	12	259	2.30–1550	98.74	65.07	16.85	63	4.69	2260.22	4.73
**Chen et al., 2012 (18)**	SNP chip (60 k)	1693	565	50.39–8102.06	252.71	247.55	139.87	147	10.94	10365.15	21.69
**Wang et al., 2013 (17)**	SNP chip (60 k)	14	63	3.20 –827.21	97.85	158.37	9.98	13	0.97	1218.39	2.55
**Rubin et al., 2012 (21)**	Genome sequencing	117	1,928	0.12–175.50	3.00	5.23	10.08	172	12.80	4422.54	9.25
**Paudel et al., 2013 (22)**	Genome sequencing	16	3,118	6.00–96.00	10.00	12.74	39.72	104	18.67	5656.82	31.74
**This study**	aCGH (1 M)	12	1,344	3.37–1319.01	11.11	35.56	47.79	—	—	—	—

Note: The comparison was based on *Sscrofa* 10.2 assembly (http://www.ensembl.org/Sus_scrofa/Info/Index). For CNVRs based on the other porcine assembly, we firstly converted the data to current genome coordinates using the UCSC LiftOver tool (http://genome.ucsc.edu/cgi-bin/hgLiftOver); CN loss counts were removed from our list while making comparison with Paudel et al., 2013 (22) as the study only reported copy number gain events.

What is worth mentioning is the comparison with one of our previous studies based on Porcine SNP60 BeadChip [17]. Though almost the same samples used in the two studies, the overlapped percentages were only 0.97% for CNVRs count and 2.55% for CNVR length, which could be explained by the limitation of the Porcine SNP60 array. Although CNV detection is also feasible with such panel, it is impaired by low marker density, non-uniform distribution of SNPs along pig chromosomes and lack of non-polymorphic probes specifically designed for CNV identification [34]. This clearly clues us that the high-density aCGH array panel can act as a considerable tool in CNV identification due to its detection power and acceptable cost.

### Quantitative real time PCR confirmation

About three-quarters CNVRs identified in the study were reported for the first time. In order to confirm these CNVRs, 21 CNVRs, representing different predicted status of copy numbers (*i.e.*, loss, gain and both) and different CNVR frequencies (varied from 8.33% to 100%), were chosen to be validated by qPCR. One or two pairs of primers (Table S3 in File S1) were designed for each CNVR and a total of 36 qPCR assays were performed. Out of the 36 qPCR assays, 28 (77.78%) were in agreement with prediction by aCGH. When counting the CNVRs, 18 (85.71%) out of the 21 CNVRs had positive qPCR confirmations by at least one PCR assay. The detail information of the confirmed 18 CNVRs was listed in Table 2. Our confirm rate was higher than or similar with previous studies [15], [16], [18], [19], [35]. Beside the high-density of the aCGH used, the main possible reason for the high confirmation rate was the stringent filter criteria as described in material and methods.

**Table 2:** Results of quantitative real-time PCR analysis of the 18 confirmed CNVRs.

CNVR#	Chr^a^	Start^a^	End	Type	Primer	Positive samples			Negative samples		
						Sample detected	Sample confirmed	Positive predictive rate	Sample detected	Confirmed samples	Negative predictive rate
**9**	1	18347414	18379506	loss	A2	3	3	1.0000	9	4	0.4444
**87**	1	284428678	284521660	loss	CGH1-1	11	11	1.0000	1	1	1.0000
					Gene2-2	11	11	1.0000	1	1	1.0000
**101**	1	296555516	296570221	loss	E1-2	2	1	0.5000	10	0	0.0000
**234**	11	62512880	62910688	both	F1-2	10	10	1.0000	2	2	1.0000
					F1-3	10	10	1.0000	2	2	1.0000
**235**	14	133945595	134212123	gain	Gene5-4	2	2	1.0000	10	1	0.1000
**240**	11	70582866	70599250	gain	J1-3	1	1	1.0000	11	8	0.7273
**296**	13	34208583	34234865	loss	CGH3-1	5	5	1.0000	7	7	1.0000
					Gene9-4	5	5	1.0000	7	7	1.0000
**393**	14	99243227	99268572	loss	CGH9-1	3	2	0.6667	9	2	0.2222
					CGH9-2	3	2	0.6667	9	2	0.2222
					CGH9-3	3	2	0.6667	9	2	0.2222
**752**	3	95978937	95991620	gain	CGH7-1	12	12	1.0000	0	0	—
**793**	4	50047123	50284182	loss	E4	5	4	0.8000	7	3	0.4286
**842**	5	9900897	9985394	gain	G7	1	1	1.0000	11	4	0.3636
**870**	5	60760998	60776722	loss	CGH8-3	2	2	1.0000	10	2	0.2000
**936**	6	72495512	72520616	gain	I2	2	2	1.0000	10	4	0.4000
				loss	I4	2	2	1.0000	10	4	0.4000
**995**	7	50302129	50673147	loss	D1-1	1	1	1.0000	11	0	0.0000
					D2-1	1	1	1.0000	11	0	0.0000
**1048**	8	4481028	4498936	loss	Gene3-4	3	3	1.0000	9	6	0.6667
**1090**	8	43313707	43894062	gain	kit6	2	2	1.0000	10	0	0.0000
					CGH2-1	2	2	1.0000	10	0	0.0000
**1322**	X	91199797	91210413	loss	CGH10-3	5	4	0.8000	7	4	0.5714
**1323**	X	91329230	91359396	loss	CGH5B-1	6	6	1.0000	6	4	0.6667
					CGH6A-2	6	6	1.0000	6	4	0.6667
								0.9321			0.4732

a The *Sus scrofa* 10.2 assembly (http://www.ensembl.org/Sus_scrofa/Info/Index) was used to indicate the position of the CNVRs.

All the 12 test samples and the reference sample in the study were tested in the qPCR assays. Consequentially, we also calculated the positive predictive rates and negative predictive rates for the 18 CNVRs confirmed by qPCR analysis. As showed in Table 2, the average positive predictive rate is 93.21%, demonstrating that, for the positive samples, qPCR assays agree well with the aCGH prediction. Contrary to the positive samples, for some of the negative samples, qPCR assays did not agree with the aCGH prediction, *i.e.* high negative predictive rate (47.32%) observed. False negative identification has been reported previously in pigs and other mammalian species [15], [16], [36]. It can be explained by the stringent criteria of CNV calling which minimize the false-positive, on the other hand lead to high false-negative rate inevitably.

### Gene content and functional annotation

Out of the total porcine genes locating in the 20 chromosomes, 1,342 porcine genes (Table S4 in File S1) were completely or partially overlapped with CNVRs, including 975 protein-coding genes, 345 pseudo genes, 7 miscRNA genes and 15 genes of other types. To test whether genes unaffected by CNVs exhibited a different selective constraint than the ones affected, we compared the dN/dS ratios for orthologous genes of pig with those of human (Table S5 in File S1). The results showed that that CNVR-related genes had dN/dS ratios significantly higher than those with normal copy numbers by Wilcoxon rank-sum tests (*P*<3.2E-16), which was consistent with the previous results in pigs and other species [20], [27]. This result indicated that, compared to genes in non-CNV regions, these genes in CNVRs might undertake a different selective constraint and be subjected to a relaxation of constraint due to the redundancy expected from the variable number of gene copies.

To provide insight into the functional enrichment of the CNVs, using the online DAVID bioinformatics resources, we also performed GO and KEGG pathway analyses for the genes in CNVRs. The GO analyses revealed 83 terms (Table S6 in File S1), of which 14 were statistically significant after Benjamini correction, while the KEGG pathway analyses revealed 12 terms (Table S7 in File S1), of which only 2 were statistically significant (Olfactory transduction and Arachidonic acid metabolism) after Benjamini correction. In accordance with previous studies in pigs and other mammals [16], [35], [37], [38], the GO analyses have evidenced that CNVRs are particularly enriched in genes related to sensory perception of the environment (sensory perception of smell and chemical stimulus, cognition), neurological system process (neurological system process), immunity (antigen processing and presentation of peptide antigen via MHC class I) and other basic metabolic processes (e.g., G-protein coupled receptor protein signaling pathway).

Chinese indigenous pig breeds and modern commercial breeds show obvious differences in many aspects, such as, growth rate, meat quality, disease resistance, sexual behavior and reproduction [39]–[41]. Previous studies have shown that CNVs play an important role in phenotypic variation and are often related with disease susceptibility [8], [42]. Thus we performed overlapping analyses of identified CNVRs with the reported QTL regions collected in the pig QTL database (Apr 20, 2013, (http://www.animalgenome.org/cgi-bin/QTLdb/SS/index)) and human disease gene orthologs in Online Mendelian Inheritance in Man annotations (OMIM, http://omim.org/, 2013-6-19). Since some QTL had too large confidence intervals, we focused on the 3,789 QTL with confidence interval less than 5 Mb. Consequentially, 446 out of the 1,344 CNVRs harbored or partially overlapped with 696 QTL (Table S8 in File S1). These QTL are involved in many traits, such as growth, meat quality, reproduction, immune capacity and disease resistance. For the human disease gene orthologs in OMIM, we found that 52 genes (Table S9 in File S1) associated with human diseases, such as Myeloproliferative disorder, Hennekam lymphangiectasia-lymphedema syndrome and Alpha-ketoglutarate dehydrogenase deficiency.

Further probing the potential functions of these copy number variable genes, we also found a suite of genes related important traits, such as coat color (v-kit Hardy-Zuckerman 4 feline sarcoma viral oncogene homolog (*KIT*)), immune response (e.g., interferon regulatory factor 2 (*IRF2*), beta-defensin 114 (*BD114*), major histocompatibility complex (*MHC*), antibacterial protein (*PMAP-23*)), sexual and reproduction ability (e.g., salivary lipocalin (*SAL1*), pregnancy-associated glycoprotein 2-like (*LOC100737419* and *LOC100524786*)), nutrients metabolism (e.g. fatty acid synthase (*FASN*), collagen alpha-4(*VI*) chain (*Col6a4*)), cationic amino acid transporter 3 (*CAT3*), the vitamin D3 25-hydroxylase (*CYP2D25*)). Considering the vital role of them, these genes provide a rich resource for testing hypotheses on the genetic basis of phenotypic variation within and among breeds, especially those between Chinese indigenous breeds and the modern commercial ones. However, due to the limited samples used in the study, the conclusion needs further research.

## Conclusions

In summary, using one custom-designed 2.1 M aCGH, we have comprehensively performed genome-wide CNV identification across the diverse pig breeds. A total of 1,344 CNVRs were identified, covering 47.79 Mb (∼1.70%) of the pig genome. In general, more CNVs per individual were identified in individuals from Chinese breeds (202.7) than in those of modern commercial breeds (109.5). Compared with previous studies, most of the CNVRs (74.18%) identified in the study were novel findings. In order to confirm these CNVRs, 21 CNVRs were chosen to be validated by qPCR and a high rate (85.71%) of confirmation was obtained. Functional annotation of CNVRs and genes involved suggested CNVRs identified have important function, and may play an important role in phenotypic and important production traits difference among various breeds.

## Supporting Information

File S1 Supporting tables. Table S1, Detailed information of each CNVR identified in this study. Table S2, Detailed information between CNVRs detected in the study with those in the previous reports. Table S3, Detailed information of the validated CNVRs and the primers used in quantitative PCR analyses. Table S4, Annotation of genes in CNVRs detected in this study. Table S5, dN/dS ratio of genes in CNVRs identified and non-CNVRs. Table S6, Gene Ontology of genes in CNVRs identified. Table S7, Pathway of genes in CNVRs identified. Table S8, QTLs harbored within or partially overlapped with identified CNVRs across the pig genome. Table S9, CNVRs completely or partially overlapped genes in Online Mendelian Inheritance in Man annotations.
(XLS)
